# Clinically Symptomatic Vitamin E-Associated Peripheral Neuropathy: An Overlooked Reversible Neurological Sequela of Exocrine Insufficiency of Chronic Pancreatitis With Type 3c Diabetes

**DOI:** 10.7759/cureus.104766

**Published:** 2026-03-06

**Authors:** Raghav Chanday, Bhavik Sethi, Saumya Ahluwalia

**Affiliations:** 1 Medicine, Dayanand Medical College and Hospital, Ludhiana, IND; 2 Internal Medicine, Apollo Hospitals, New Delhi, IND; 3 Gastroenterology and Hepatology Research, Mayo Clinic Alix School of Medicine, Rochester, USA

**Keywords:** chronic pancreatitis, exocrine pancreatic insufficiency, malabsorption, pancreatic exocrine dysfunction, peripheral neuropathy, sensorimotor polyneuropathy, steatorrhea, type 3c diabetes mellitus, vitamin e deficiency, α-tocopherol deficiency

## Abstract

Diabetes mellitus (DM) after an episode of chronic pancreatitis, known as type 3c diabetes, is becoming an exceedingly common entity. Not only endocrine but also chronic pancreatitis-associated exocrine pancreatic insufficiency (EPI) is being increasingly described. After pancreatitis, EPI usually manifests as malabsorption with steatorrhea detected by fecal elastase; a high index of suspicion is required to diagnose exocrine sequelae. In fact, clinically symptomatic vitamin E deficiency is especially rare and almost overlooked and underrecognized. Furthermore, subclinical fat-soluble vitamin E deficiency due to EPI is seldom recognised. We report a case of chronic pancreatitis-related sensorimotor peripheral neuropathy due to vitamin E deficiency, which resolved on administration of vitamin E. To the best of our knowledge, chronic pancreatitis-related EPI fat-soluble vitamin E deficiency-associated neurological sequelae have been overlooked and rather neglected in medical literature. In diabetic patients, neuropathy-related symptoms are always clinically attributed as a complication of diabetes per se.

## Introduction

Diabetes mellitus (DM) developing after chronic pancreatitis is classified as type 3c diabetes mellitus (T3cDM), also known as pancreatogenic diabetes, which results from structural damage to the pancreas leading to impaired insulin secretion [1]. In addition to endocrine dysfunction, patients with chronic pancreatitis often develop exocrine pancreatic insufficiency (EPI), a condition characterized by inadequate production of digestive enzymes necessary for nutrient absorption. As a result, patients may develop malabsorption, most commonly presenting as steatorrhea, defined as bulky, foul-smelling, fatty stools due to impaired fat digestion. EPI is often screened using Sudan staining of feacal fat and feacal elastase, a non-invasive stool test that measures pancreatic enzyme output. Low levels indicate exocrine dysfunction [2,3].

Fat malabsorption in EPI predisposes patients to deficiencies of fat-soluble vitamins (A, D, E, and K). Vitamin E (α-tocopherol), a lipid-phase antioxidant, plays a critical role in protecting neuronal cell membranes from oxidative damage. Deficiency may result in large-fiber sensorimotor polyneuropathy characterized by loss of vibration and proprioception, ataxia, and areflexia. However, in patients with concurrent T3cDM, neurological symptoms are invariably ascribed to diabetic neuropathy, often delaying recognition of vitamin deficiency as an alternative and potentially reversible cause of polyneuropathy. In fact, methodical evaluation of all possible differentials of reversible neuropathy is of paramount importance to establish a definitive causal association.

We report a case of chronic pancreatitis-related sensorimotor peripheral neuropathy secondary to vitamin E deficiency in the setting of EPI. The temporal correlation between vitamin E supplementation and marked neurological improvement and the absence of other reversible causes confirmed vitamin E deficiency as a likely contributor to neuropathy. Since the patient also had associated T3cDM, the neurological manifestations were initially attributed to diabetic neuropathy. To the best of our knowledge, vitamin E-associated neurological sequelae specifically in patients with pancreatitis-related EPI have not been adequately emphasized in the medical literature, with only occasional mention of this entity [4]. Our case highlights the need for clinicians to recognize pancreatic polyneuropathy as a potentially reversible complication of exocrine dysfunction, underscoring the importance of simple evaluation of vitamin E levels and timely supplementation to mitigate disabling pancreatic neuropathic symptoms significantly affecting quality of life (QOL) in this subset of patients.

## Case presentation

A 34-year-old gentleman presented in the out-patient clinic with complaints of numbness and tingling in the bilateral upper and lower extremities for two months. He noted slight lower extremity weakness and complained of difficulty in getting up from supine position. He also had been complaining of diarrhea since the last one year, with five to six bulky stools daily. He had noticed that the diarrhea frequency significantly reduced on reducing ­food intake. There were no other constitutional or neurological symptoms. The patient did not have any pertinent history on medication reconciliation. The patient also gave history of chronic moderate alcohol consumption up to 30-60 mL/day for eight years and had abstained from alcohol following a pancreatitis episode five years back. During follow-up evaluation, he was diagnosed with T3cDM after one year of the episode and was started on oral hypoglycemic agents. Six months later, he was switched to insulin due to uncontrolled glycemic status and achieved good glycemic control (HbA1c 6.4%).

General physical examination was unremarkable. Neurological examination revealed mild generalised muscle wasting with normal tone. The patient had bilateral 4/5 power in hip flexion and adduction with similar weakness (4/5 power) in plantar flexion and extension at both ankle joints. All other muscles had normal power. Deep tendon reflexes in the lower limbs were normal except for the bilateral absent ankle reflex. There was a flexor plantar response. Sensory examination showed impaired vibration till mid-tibia with impaired proprioception at the metatarsophalangeal joint. Fine touch, pin prick, pain, and temperature were relatively normal bilaterally. 

**Figure 1:**
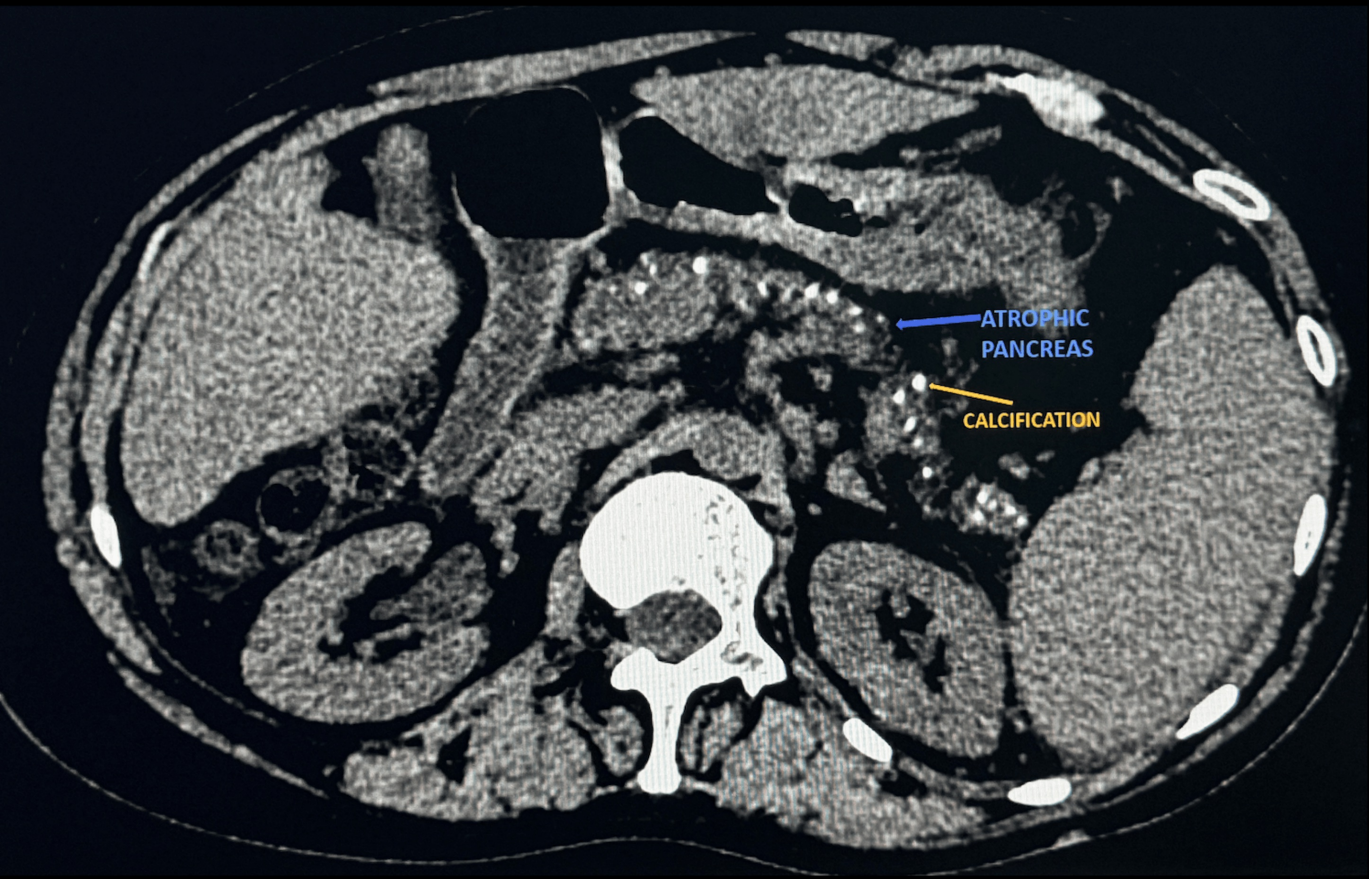
Axial non-contrast computed tomography (NCCT) revealed atrophic pancreas (blue arrow) with specks of parenchymal calcification (yellow arrow) suggestive of chronic calcific pancreatitis Pancreatic atrophy reflects long-standing parenchymal destruction and fibrosis, while coarse parenchymal calcifications (hyper intensities marked by yellow arrow) are characteristic of chronic calcific pancreatitis, resulting from repeated inflammation and ductal obstruction leading to protein plug formation and calcification. These imaging findings corroborate the diagnosis of advanced chronic pancreatitis with exocrine insufficiency.

Routine laboratory evaluation revealed undetectable serum lipase, normal B12 levels, and a deranged prothrombin time corrected after vitamin K administration. This raised the suspicion of the conventional fat-soluble vitamin K deficiency. Vitamin A, D, and folate levels were not assessed. Weight loss was absent and no changes in appetite were expressed by the patient. Liver function tests and thyroid studies were unremarkable. No relevant family history of diabetes, hypertension, or alcoholic liver disease was present. Patient reported abstinence from alcohol and smoking for the past five years. Diarrhea evaluation revealed positive Sudan staining of faecal fat, suggestive of steatorrhea. Additionally, though fecal elastase testing is the gold standard, it could not be performed due to resource limitations. A nerve conduction study was suggestive of predominantly sensorimotor neuropathy with no conduction defects. Diabetic neuropathy was particularly ruled out considering the patient had persistent symptoms of neuropathy despite optimal glycemic control (HbA1c 6.4%), absence of length-dependent axonal polyneuropathy on nerve conduction study, and dramatic improvement after vitamin E supplementation. Non-contrast computed tomography (NCCT) abdomen revealed atrophied pancreas with features suggestive of chronic calcified pancreatitis (Figure 1), thereby confirming the laboratory evaluation of pancreatic insufficiency.

In view of intractable neurological symptoms affecting his QOL with good glycemic control (HbA1c 6.4%), evaluation of vitamin E deficiency as a rare causative factor was considered and evaluated. After ruling out pertinent differentials for neuropathy after a multitude of laboratory tests, our index of clinical suspicion of vitamin E deficiency was confirmed with tocopherol levels 1.72 mg/L (normal 5.0-18.0 mg/L) by high-performance liquid chromatography methodology (Table 1). The patient was initiated on oral vitamin E (α-tocopherol) supplementation of 400 mg daily, with the patient reporting significant resolution of symptoms after four weeks of follow-up. His repeat tocopherol levels were 10.12 mg/L (normal 5.0-18.0 mg/L). Also, the potential benefits of pancreatic enzyme replacement therapy (PERT), which is the cornerstone in EPI management to prevent recurrence of EPI complications, were discussed with the patient. Unfortunately, in view of financial limitations, the patient declined PERT.

**Table 1:** Laboratory report for vitamin E levels

Test Name	Result	Bio Ref Interval
Vitamin E; Tocopherol, Serum (HPLC)	1.72	5.00 - 18.00
REFERENCE RANGES		
Reference Group	Reference Range (mg/L)	
Premature infants	1–5	
1–12 years	3–9	
13–19 years	6–10	
Adults	5–18	
CRITERIA		
Criteria	Levels of Vitamin E (mg/L)	
Significant deficiency	<3	
Significant excess	>40	

## Discussion

This case highlights the need for a high index of suspicion to assess a concomitant micronutrient deficiency in patients of chronic pancreatitis with T3cDM who present with overt neurological manifestations of polyneuropathy not improving with good glycemic control. Although both EPI and T3cDM are well characterized sequelae of pancreatitis, polyneuropathy symptoms tend to be ascribed to diabetes [1-3]. In fact, despite significant cases of EPI after chronic pancreatitis being attributed to subnormal vitamin E levels, clinically symptomatic neuropathy attributed to the same is exceedingly rare [4,5]. Vitamin E (α-tocopherol), being a potent lipid-phase antioxidant, protects neuronal cell membranes from oxidative damage. Its deficiency results in selective injury of large myelinated axons, producing a polyneuropathy characterized by loss of vibration and proprioception, ataxia, and areflexia [6].

In our patient, the presence of steatorrhea, undetectable serum lipase, and pancreatic atrophy with calcification confirmed EPI as the underlying aetiology of malabsorption. Normal B12 levels, unremarkable liver function tests and thyroid studies, negative medication reconciliation history, and low serum tocopherol levels established vitamin E as the probable biochemical diagnosis of intractable polyneuropathy, and the rapid clinical improvement following vitamin E supplementation with normalisation of vitamin E levels reinforced vitamin E as a possible contributor to neuropathy. Thus, clinicians need to think with an out-of-the-box clinical approach when clinical symptoms do not improve with the conventional approach. However, as a word of caution, clinical wisdom dictates that common causes first, and thereafter the uncommon and rare causes should be considered when patients don’t improve and not vice versa! In this case, neurological symptoms were attributed to the very common diabetes-related glycosylation neuropathic injury by clinicians taking care of the case earlier, and rightly so because complications of DM would be the commonest etiology.

Our evaluation focused on soft clues in the history of no improvement with well-controlled glycemic status after switching to insulin and EPI symptoms. Our case highlights the need for a high clinical index of suspicion to evaluate for fat-soluble vitamin deficiency, as EPI evaluation with vitamin E is an important possible etiology of reversible polyneuropathy. The present case adds to the insufficient medical literature by linking chronic pancreatitis-related EPI, T3cDM, and neurological sequelae, and thus highlights reversible causes of polyneuropathy in patients with exocrine-endocrine sequelae of chronic pancreatitis.

## Conclusions

Our case highlights the need of a high clinical index of suspicion to evaluate for fat-soluble vitamin deficiency due to EPI in patients with reversible polyneuropathy especially in the setting of concomitant T3cDM and chronic pancreatitis-related EPI. The present case adds to the insufficient medical literature by linking chronic pancreatitis-related EPI, T3cDM, and neurological sequelae - a triad that underscores the exocrine-endocrine component of pancreatic disease and allows timely supplementation and prevention of irreversible neurological damage.
